# Clinico-genetic heterogeneity in Pakistani families affected with muscular dystrophies

**DOI:** 10.1007/s11033-026-11679-0

**Published:** 2026-03-19

**Authors:** Riaz Ahmad, Muhammad Almas Hashmi, Asad Ullah, Ubaid Ur Rehman, Muhammad Naeem, Henry Houlden

**Affiliations:** 1 Medical Genetics Research Laboratory, Department of Biotechnology, Quaid-i-Azam University, Islamabad, 45320 Pakistan; 2 Department of Neuromuscular Disorders, UCL Queen Square Institute of Neurology, Queen Square House, London, WC1N 3BG UK

**Keywords:** MDC1A, *LAMA2*, *DMD*, Next-generation sequencing, Novel variant

## Abstract

**Background:**

Muscular dystrophies are a group of inherited neuromuscular disorders characterized by degenerative and progressive muscle weakness. Duchenne muscular dystrophy (DMD), Becker muscular dystrophy (BMD) and merosin-deficient congenital muscular dystrophy type 1 (MDC1A) are frequent forms caused by *DMD* (NM_004006.3) and *LAMA2* (NM_000426.4) mutations, respectively.

**Methods and results:**

Our study used whole exome sequencing to explore the molecular genetics of muscular dystrophies in six unrelated Pakistani families. We found four novel variants in the *LAMA2*; c.7470_7473del; p.(Lys2490AsnfsTer56) in family A, c.7807del; p.(Ala2603HisfsTer4) in family C, c.8651T > C; p.(Met2884Thr) in family D and c.4127T > A; p.(Leu1376Ter) in family E associated with MDC1A in these families. Furthermore, two already cited *DMD* variants were identified: c.10,801 C > T; p.(Gln872Ter) in family B and hemizygous genomic deletion in *DMD* (NM_004006.3):g.32438241_32809611del (corresponding to deletion of exons 7–29; GRCh38), which was identified (in family F) associated with BMD and DMD, respectively.

**Conclusions:**

We present the first report of MDC1A-associated phenotypes caused by the *LAMA2* gene in the Pakistani population, while *DMD*-related cases have already been documented in the literature and public databases. Clinical and molecular diagnosis of these six affected families will be valuable in therapeutic interventions and prenatal diagnosis of neuromuscular dystrophies in the familial and Pakistani populations. Our study emphasizes the effectiveness of next generation sequencing technology over traditional diagnostic methods such as muscle biopsies.

## Introduction

Muscular dystrophies (MDs) are a group of inherited degenerative neuromuscular disorders that primarily manifest as progressive muscle weakness, leading to significant morbidity. Now, a total of nine specific classes of muscular dystrophy have been diagnosed because of mutations in 57 genes [1]. Duchenne muscular dystrophy (DMD) is the most prevalent and severe form of muscular dystrophy and is caused by mutations in the *DMD* (dystrophin) (ENST00000357033.9) gene, which is located on the X chromosome [2]. DMD affects approximately 1 in 3600 males [3]. DMD is clinically marked by muscle weakness and atrophy during early childhood that begins by the age of 3 years. Early manifestations include pain or difficulty with walking, delayed growth, a waddling gait and frequent falls [4]. Most affected individuals lose ambulation during late childhood or adolescence and die in early adulthood [5, 6]. The second common form of muscular dystrophy is Becker muscular dystrophy (BMD), which is also caused by variants in the *DMD* gene and has a global prevalence of approximately 1.5–3.6 in 100,000 males. The milder BMD form is investigated in patients around 11 to 25 years of age; however later age of onset is possible. BMD is characterized by progressive weakness in the shoulders, thighs, hips and pelvis muscles. Patients of BMD have nearly normal lifespans unless they experience cardiac arrest [7].

Merosin-deficient congenital muscular dystrophy type 1 (MDC1A), or laminin-α2chain-deficient congenital muscular dystrophy, caused by variants in the *LAMA2* gene (ENST00000421865.3)and inherited in an autosomal recessive manner, is one of the most common subtypes of congenital muscular dystrophy (CMD). In MDC1A, the expression of laminin-α2chain is either reduced or completely absent, leading to early-onset clinical manifestations such as muscle weakness, profound hypotonia, skeletal deformities, inability to achieve ambulation and respiratory complications [8].

Congenital muscular dystrophies (CMDs) are rare disorders with an incidence of 0.82 per 100,000 live births [8]. The most frequent types are laminin-α2chain, collagen type VI and alpha-dystroglycan related types. As reported by Zambo and Muntoni, 37 genes have been associated with congenital muscular dystrophies [9]. The absence or deficiency of the laminin-α2 subunit results in the loss of laminin-211 and/or laminin-221, leading to compromised stability and strength of tissues in skeletal muscle. Depending on the degree of laminin-α2 deficiency, the clinical manifestations range vary from severe MDC1A (OMIM: 607855) to mild late onset *LAMA2*-MD (OMIM: 618138). In severe MDC1A, newborns are characterized by a weak cry, hypotonia and muscle weakness, which lead to delayed motor developmental milestones [10–11]. Only a few children can walk with support; otherwise, most are non-ambulatory [12]. Other complications include difficulty in feeding, swallowing and chewing because of restricted growth [13]. Several patients require enteral feeding and may also develop respiratory problems and occasionally need ventilatory support during their lifetimes [14]. In 30% of the patients, death is caused by respiratory tract infection (RTI) in the first decade of life with early onset of MDC1A [15]. In low- and middle-income countries (LMICs), mostly patients remain misdiagnosed or undiagnosed. The genetic architecture of muscular dystrophies varies across populations, and in regions with high consanguinity, such as Pakistan, autosomal recessive forms of inherited neurological disorders are more common [16]. Limited molecular data of muscular dystrophies from Pakistan have been published, leaving the population’s mutational spectrum poorly characterized, hindering accurate diagnosis, genetic counseling, and potential therapeutic interventions.

This study aimed to use exome sequencing as a first line of diagnostic approach to identify the cause of the disease. Through NGS technology we investigated six unrelated Pakistani families affected with mild to severe manifestations of muscular dystrophies. Disease-causing variants were identified in these families that strengthen the genotype-phenotype correlation and explore the disease burden around the globe. Early diagnosis and genetic counseling help in reducing the impact and mortality of the disorder.

## Materials and methods

### Ethical approval, recruitment and human subjects

Six unrelated human families were recruited from different regions of Pakistan including Khyber Pakhtunkhwa Province, Punjab Province and Islamabad Capital Territory. The study was approved by the Institutional Review Board of Quaid-I-Azam University, Islamabad, Pakistan (Approval No. QAU/DFBS/216). Written informed consent was obtained from the parents or legally authorized representatives of all minor subjects included in this study. Blood samples were collected from the participating normal and affected individuals of the families. Genomic DNA was extracted from peripheral blood lymphocytes by standard extraction protocol.

### Exome sequencing and variants calling

Whole exome sequencing for six families (A, B, C, D, E and F) was performed at Macrogen, Korea through the Agilent SureSelect Human All Exome V6 Kit (Agilent Technologies, Santa Clara, CA, USA) as described by Efthymiou et al. [17] and Ahmad et al. [18]. Paired-end sequencing or PE150 was done by Illumina NovaSeq 6000 (Illumina, Santa Clara, CA, USA). The resultant read of sequencing against the reference genome of humans was aligned through Burrows-Wheeler Aligner v0.7.17 (BWA). Human genome assembly (hg19) was selected for our enrolled families, and hg38 assembly was used for sequencing reads. BAM files were sorted duplicated reads were identified via SAMtools (v1.8) and Picard (v2.18.9), respectively. Genome Analysis Toolkit (GATK) v4.0 was used for genotyping. Further, functional annotation and variant filtration were done by Annotate Variation (ANNOVAR) and FILTUS, respectively. After annotation, the obtained file was recovered in CSV format which was further filtered to identify the most accurate possible pathogenic variants. Our bioinformatic filtration strategy focused on searching for the exonic region (coding region), splice donor and acceptor site as described previously in supplementary Figure S1 [19]. Following pedigrees and clinical history, we chose the rare variants with MAF < 0.01% in databases including 1000 Genomes project, NHLBI Exome Variant Server, Exome Aggregation Consortium and Complete Genomics 6. Inheritance patterns and different in silico tools (shown in Table 1) were also considered for nonsense, missense, splice site and frameshift mutations. Pathogenic and likely pathogenic variants were prioritized based on ACMG guidelines. Our phenotypes are associated with muscular dystrophies so copy number variations (CNVs) analysis was also checked accordingly.

**Table 1:** Genetic findings of six unrelated families associated with muscular dystrophies in this study

Family ID: (patient ID)	Gene	Genomic position	cDNA HGVS	Protein HGVS	Mode	Type of variant	Allelic Frequencies	CADD Score	dbSNP ID	Mutation Taster	Polyphen-2	Reference	ACMG
A: (IV:3)	*LAMA2*	Chr6:129478708	c.7470_7473del	p.Lys2490AsnfsTer56	AR	Frameshift	NA	NA	NA	NA	NA	Novel	LP
B: (III:1)	*DMD*	ChrX:31,146,411	c.10,801 C > T	p.Gln872Ter	X-linked	Nonsense	NA	45	NA	Disease-causing	NA	Reported	LP
C: (III:1)	*LAMA2*	Chr6:129486527	c.7807del	p.Ala2603HisfsTer4	AR	Frameshift	NA	NA	NA	NA	NA	Novel	LP
D: (III:1)	*LAMA2*	Chr6:129505303	c.8651T > C	p.Met2884Thr	AR	Missense	Exomes: ƒ =0.00000479	19.35	rs772282706	Disease-causing	Possibly damaging	Novel	VUS
E: (IV:3)	*LAMA2*	Chr6:129320606	c.4127T > A	Leu1376Ter	AR	Stop gained	NA	38	NA	Disease-causing	NA	Novel	P
F: (III:3)	*DMD*	ChrX: 32438241–32809611	c.32438241-32809611del	NA	X-linked	Gross deletion	NA	NA	NA	NA	NA	Reported	P

NA: Not available, LP: Likely pathogenic, P: Pathogenic, VUS: Variant of unknown significance, AR: Autosomal recessive

### Homozygosity mapping

**Fig. 1:**
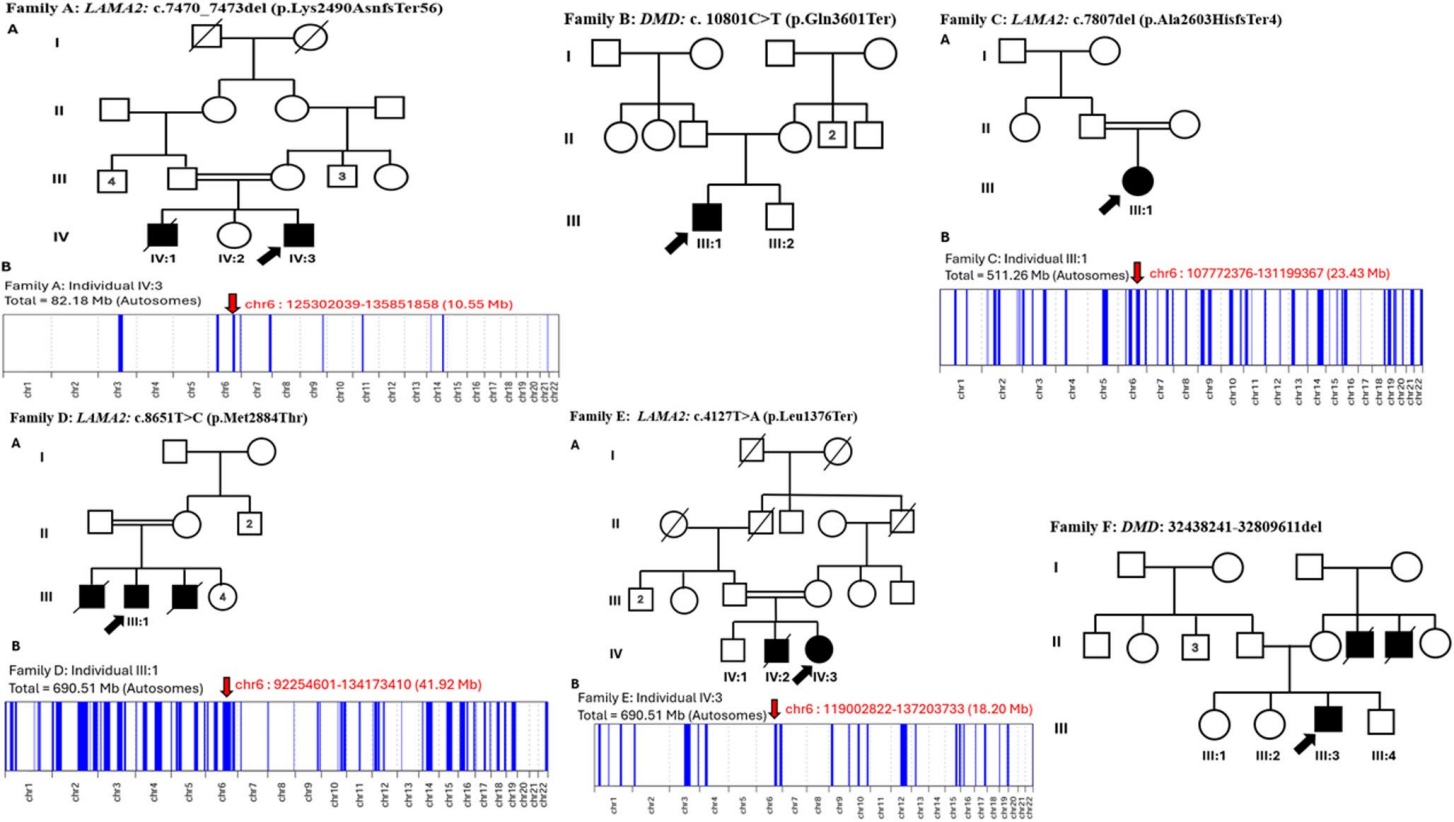
Pedigree and homozygosity mapping of six unrelated families (A, B, C, D, E and F). Probands subjected to whole exome sequencing are denoted by arrows. Squares represent males, while circles represent females. Unfilled symbols indicate healthy subjects, and filled symbols indicate affected individuals. Consanguinity is signified by a double line. Homozygosity mapping was performed for families A, C, D and E, with the target region was highlighted by a red arrow in the respective families

To confirm the homozygous region shared by ancestors, we performed homozygosity mapping through the AutoMap (https://automap.iob.ch/process), which was used with default settings (as shown in Fig. 1 for families A, C, D and E). VCF (Variant Call Format) files of WGS or WES could be used to generate homozygosity stretches [20].

### Prediction programs

The mutational effect was checked for all missense variants using the CADD score (Combined Annotation Dependent Depletion (CADD; https://cadd.gs.washington.edu), MutationTaster (www.mutationtaster.org) and PolyPhen-2 (http://genetics.bwh.harvard.edu/pph2). Identified variants were further classified as likely pathogenic and pathogenic according to ACMG criteria using the Franklin variant assessment tool (https://franklin.genoox.com/clinical-db/home) and VarSome (https://varsome.com/).

## Results

The genetic diagnoses and clinical findings of the patients are listed in Tables 1 and 2, respectively.

**Table 2:** Clinical findings of affected families with diverse muscular dystrophies

Clinical features	Family A	Family B	Family C	Family D	Family E	Family F
Patient or proband ID	**IV:3**	**III:1**	**III:1**	**III:1**	**IV:3**	**III:3**
Likely OMIM syndrome: OMIM ID	MDC1A: OMIM (607855)	BMD: OMIM (300376)	MDC1A: OMIM (607855)	MDC1A: OMIM (607855)	MDC1A: OMIM (607855)	DMD: OMIM (310200)
Gender	M	M	F	M	F	M
Current age (year)	17 years	19 years	13 years	10 years	5 years	16–17 years
Disease onset	By birth, a weak cry	Late-onset (5 years)	3 months	1 year	By birth (delayed crying)	(3 years)
Consanguinity	Yes	No	Yes	Yes	Yes	No
Progressive	Yes	Yes	Yes	Yes	Yes	Yes
Facial dysmorphy	Elongated myopathic face	No	No	Yes	Milder	Milder
Feeding difficulties	Yes	No	Yes	NA	Yes	Yes
Hypotonia	In both legs	Yes	Yes	Yes	Yes	Yes
Musculature	Muscle weakness in lower and upper limbs	Yes	Yes	Yes	Yes	Yes
Creatine kinase level	High (1080 U/L)	High (1255 U/L)	NA	NA	High (902 U/L)	High (8200 U/L)
Contracture	Yes	Yes	Yes	Yes	Yes	Yes
Scoliosis	Yes	NA	NA	NA	Yes	Yes
Speech impairment	Yes	Mild	Yes	No	Yes	Yes
Respiratory complication	Yes	Yes (Repeated chest infections)	NA	Yes	Yes	Yes
Loss of Ambulation	2-3 years	15 years of age	Yes	NA	Yes	8-9 years
Intellectual disabilities	Yes	Mild	Yes	Moderate	Yes	Yes
Seizures	Fibrile seizures	No	NA	No	No	No

### Family A

Subject (IV:3) presented with congenital muscular dystrophy characterized by early onset with a weak cry. The patient was 17-year-old at the time of the study. Clinical features include an elongated, myopathic facial appearance, feeding difficulty, joint contractures, hypotonia of the lower limbs, spinal curvature or scoliosis and significant progressive muscle weakness of the proximal limbs. The patient has muscle wasting, joint immobility and developmental milestones delay and the child never walks on his own. The condition caused feeding difficulties because of hypotonia, with the possibility of dependency on enteral feeding. The patient has stunted growth while the overall motility of the patient is significantly affected. During winter, the proband’s respiratory complications tend to worsen, mostly leading to hospitalization. His 5-year-old brother had died because of the same condition. Clinical phenotype and molecular analysis confirmed our case correlated to MDC1A.

### Family B

The patient (III:1) is a 19-year-old male who started developing slow progressive walking impairment at the age of 5 years. The patient started walking at an age of 1.5 years, neck holding at 9 months and sitting at 1 year of age. The subject exhibits a positive pseudohypertrophy and Gower sign – a clinical manifestation of proximal muscle weakness. Muscles of the pelvic girdle and lower limb are particularly weak. Over the last 11 years, the patient has shown a gradual deterioration in mobility during activities involving large muscle groups, such as climbing stairs or getting up from a seated position. A positive Gower sign indicates worsening muscle strength in the hip and thigh, which are vital for supporting the body and enabling movement. Weakness was gradual, progressive and bilateral. Other symptoms are summarized in Table 2. Clinicians suggested physical therapy, orthopaedic support and possible pharmacological intervention to reduce further functional decline. Family B phenotypes are closely associated with a mild type of muscular dystrophy, such as BMD.

### Family C

Subject (III:1) is a female (13 years old) and the onset of congenital muscular dystrophy happened at 3 months of age; early clinical features included significant, persistent hypotonia, lower and upper limbs abnormalities and evidence of severe delay in motor development. Other clinical details are summarized in Table 1. All these manifestations are matched with MDC1A. Family history was unremarkable for this kind of muscular dystrophy and no evidence of this condition was observed in other members.

### Family D

The proband (III:1) is a 10-year-old male (weight: 20 Kg; Height: 125 cm) born to healthy parents and presents with severe intellectual disabilities, progressive muscle weakness, low-set prominent ears, triangular face, deterioration in academic performance, and developmental delay. Cardiac and respiratory-related complications were observed. The family history is significant for two deceased siblings; one early neonatal death and another who died at 10 days of life due to prematurity. Elder male sibling with neuropsychiatric issues, while his four sisters are alive and healthy. Chromosomal or karyotyping analysis was observed normal. No speech impairment and seizures were observed in family D. All symptoms are consistent with MDC1A.

### Family E

A 5-year-old female (IV:3) was born with delayed crying and hypotonia. Birth parameters and even neonatal adaptation were not normal. She has been presented with severe muscle atrophy and has developmental disabilities of an extremely severe degree. Her family history reveals the expiration of a brother to the same condition that implied. Intellectual functioning was abnormal with no seizures. During her last assessment at the age of 5 years, speech impairment and intellectual disabilities were predominantly noticed. Based on genetic and clinical features (Table 2) proband was diagnosed with MDC1A.

### Family F

Subject (III:3) is a 14-year-old male who shows hypotonia, hyporeflexia, flexion contractures, increased lordosis, scoliosis, waddling gait, positive Gowers sign, short stature and mild mental retardation with developmental delay before 5 years of age. He was born normal to healthy parents following an unremarkable pregnancy. At a recent assessment, he is unable to stand or walk and is bound to a wheelchair. He could not speak a single word due to muscle weakness. The condition becomes worse because of the hip flexors and hamstrings. In the same family, two male patients have died because of the same condition within 20 years of life. Karyotyping analysis was done at the age of 5 years but confirmed normal for chromosomal abnormalities. Physical therapy has been conducted but no improvements have been noted.

### Genetic analysis

We studied six unrelated families that exhibited overlapping features of muscular dystrophies, including MDCA1 (Family A, C, D and E), Becker muscular dystrophy (Family B) and Duchenne muscular dystrophy (Family F). These families harbor confirmed pathogenic and likely pathogenic variants in the *LAMA2* (Human (GRCh38.p14) and the *DMD* genes. We performed WES for all families and found two novel frameshift variants c.7470_7473del; p.(Lys2490AsnfsTer56) in family A, c.7807del; p.(Ala2603HisfsTer4) in family C, one novel missense c.8651T > C; p.(Met2884Thr) in family D and one nonsense c.4127T > A; p. (Leu1376Ter) (in family E in the *LAMA2* gene. Furthermore, a nonsense variant, c. 10801 C > T; p.(Gln872Ter), was detected in family B, whereas a large genomic deletion (chrX:32438241–32809611del; corresponding to the deletion of exons 7–29) involving the *DMD* gene was identified in family F by multiplex ligation-dependent probe amplification (MLPA).

## Discussion

According to our knowledge, *LAMA2* variants associated with congenital muscular dystrophy have not previously been reported in the literature from Pakistan. This suggests that our findings expand the clinical and mutation spectrum for the diagnosis of MDC1A. *DMD* gene-based variants are cited from Pakistan for *DMD*-related disorders [21–23].

As a diagnostic method, DNA sequencing or NGS technology is recommended by different groups for neuromuscular disorders such as MDC1A because of overlapping phenotypes and genetic heterogeneity. On the other hand, the muscle biopsy method has more complications than DNA sequencing. An autosomal recessive disorder MDC1A (MIM: 607855), is caused by homozygous or compound heterozygous variations in the *LAMA2* gene [24]. The most common pathogenic mutations in *LAMA2* are frameshifts with a frequency of 82.5% [25]. The *LAMA2* gene comprises 65 exons located on chromosome 6q22-q23 [26]. Laminin alpha-2 is also known as merosin, an extracellular matrix protein that plays a crucial role in forming a connection between the extracellular matrix and cytoskeleton [27]. It is expressed in the striated muscle basement membrane, Schwann cells and basal lamina of the cerebral blood vessels [28, 29]. There is a significant variation in the clinical presentation of this disorder based on the age of onset, with most early-onset patients demonstrating severe phenotypes compared to late-onset cases. However, predicting the defined clinical manifestations based on the level of expressed laminin alpha-2 protein remains uncertain [30]. In early-onset cases, affected individuals typically present with muscle weakness, hypotonia, loss of independent ambulation, respiratory complications, proximal joint contractures and normal intellectual disabilities. In contrast, late-onset patients are characterized by proximal muscle weakness, delayed motor milestones and eventual loss of mobility [10].

In our study, we found two novel frameshift variants, c.7470_7473del; p.(Lys2490AsnfsTer56) and c.7807del; p.(Ala2603HisfsTer4), in family A and family C, respectively. Loss of function (LOF) mutations, such as frameshifts and nonsense, affect mRNA and resulting proteins that cause disease phenotype [25]. *LAMA2*-related LOF variants have pathogenic effects because of decreased levels of mRNA due to nonsense-mediated RNA decay [31]. Most reported variants in the *LAMA2* gene create premature termination codons (PTCs), as in our patients [32]. Classic CMD1A or a more severe and early-onset form of congenital muscular dystrophy is caused by both defective alleles [33]. Mild and late-onset forms of the disorder have commonly been accompanied by missense variants, which are documented in a small number of cases. These cases are correlated with partial deficiency of laminin-α2 as confirmed with muscle biopsies-based immunohistochemical tests [34]. The phenotypes in family D are less severe than those with frameshift mutations (families A and C).

A brief comparison with published cohorts further contextualizes our results. In a large UK cohort of 51 MDC1A patients, complete merosin deficiency was associated with earlier onset, lack of independent ambulation, and increased need for ventilatory and enteral support [14], supporting the correlation between truncating variants and severe congenital phenotypes observed in our study. Similarly, an international MRI-based cohort (27 patients) demonstrated a clinical continuum from non-ambulant congenital cases to milder ambulant forms, with truncating and splice-site variants accounting for 65% of mutations [35]. In contrast, the Western Sicily Limb–Girdle muscular dystrophy (LGMD) cohort reported *LAMA2* mutations in 12.5% of LGMD cases, typically with later onset, ambulant course, and lower CK levels [36]. Overall, these data highlight the broad phenotypic spectrum of *LAMA2*-related disorders and support the value of NGS in refining genotype-phenotype correlations among diverse populations.Biallelic nonsense variants exhibit variable clinical phenotypes and laminin-α2 absence can achieve independent ambulation [14]. We diagnosed family E with a novel homozygous c.4127T > A;p.(Leu1376Ter) nonsense variant with diverse clinical symptoms (Table 2).

Laminins are essential glycoproteins that play a key role in the architecture and function of the basement membrane, binding cell surface receptors via their laminin G-like (LG) domains [34]. Our variant is located in the LG5 domain together with the LG4 domain, these are the binding sites for α-dystroglycan and heparin. Therefore, this nonsense variant may affect the unions of molecules that bind the sarcolemmal cytoskeleton and extracellular matrix, ultimately disturbing the stability of laminin-α2 [14, 37]. The most common muscular disorder in childhood, Duchenne muscular dystrophy, is an X-linked recessive disorder mostly caused by deletions and duplications in the *DMD* gene. However, 10–15% of cases are caused by nonsense mutations [38]. Bitetti et al. (2021) reported a nonsense variant c.10,801 C > T; p.(Gln3601X) in the 76th exon of a 3-month-old baby from an Italian family who was a confirmed case of DMD. In family B, we report the same stop-codon point mutation as mentioned by Bitetti in a 19-year-old boy who clinically presented with hypotonia, musculature and mild speech impairment [39]. Our case was closely associated with documented cases of Duchenne muscular dystrophy. Furthermore, in family F, a gross deletion (chX: 32438241-32809611del) was observed, consistent with clinical symptoms of Duchenne muscular dystrophy. Thousands of different variations in *DMD* have been reported in BMD or DMD disorder [7, 40]. Around 60–70% of mutations in DMD patients are deletions, 5–15% are duplications and approximately 20% are point mutations, small insertions or deletions [7, 41]. By contrast, in BMD patients 60–70% of variations are deletions, 20% duplications, and 5–10% point mutations and small indels [7, 42, 43]. Several exons are considered hotspots, such as 3–19 (7%) and 45–55 (47%) for deletions in *DMD* [44, 45].

## Conclusion

For the first time, we have elucidated *LAMA2*-related MDC1A phenotypes in the Pakistani population and underscored the diagnostic utility of next-generation sequencing in the clinical and molecular diagnosis of neuromuscular dystrophies, thereby facilitating targeted therapeutic strategies and informed prenatal interventions. In Pakistan like country, translating scientific breakthroughs into public health initiatives has been challenging despite the significant impact of neurological disorders on families and patients due to the lack of a national framework for incorporating genomics into healthcare. Medical infrastructure is insufficient for even infectious diseases, so genetic health services are ignored. Factors like religion, culture and society could shape the perceptions of genetic diagnosis in low and middle-income countries. Therefore, based on limited therapeutic options for inherited neurological disorders, prenatal diagnosis, carrier testing and genetic counseling should be encouraged in early diagnosis and prevention. Genomic research should be expanded for scientific interventions across the globe.

## Data Availability

No datasets were generated or analysed during the current study.

## References

[1] Benarroch L, Bonne G, Rivier F, Hamroun D (2023) The 2023 version of the gene table of neuromuscular disorders (nuclear genome). Neuromuscul Disord 33(1):76–117 36697115 10.1016/j.nmd.2022.12.002

[2] Mah JK, Korngut L, Dykeman J, Day L, Pringsheim T, Jette N (2014) A systematic review and meta-analysis on the epidemiology of Duchenne and Becker muscular dystrophy. Neuromuscul Disord 24(6):482–491 24780148 10.1016/j.nmd.2014.03.008

[3] Yiu EM, Kornberg AJ (2015) Duchenne muscular dystrophy. J Paediatr Child Health 51(8):759–764 25752877 10.1111/jpc.12868

[4] Jennekens FG, Kate T, De Visser LP, M. and, Wintzen AR (1991) Diagnostic criteria for Duchenne and Becker muscular dystrophy and myotonic dystrophy. Neuromuscul disorders: NMD 1(6):389–391 10.1016/0960-8966(91)90001-9 1822350

[5] Shirokova N, Niggli E (2013) Cardiac phenotype of Duchenne muscular dystrophy: insights from cellular studies. J Mol Cell Cardiol 58:217–224 23261966 10.1016/j.yjmcc.2012.12.009 PMC3615054

[6] Klingler W, Jurkat-Rott K, Lehmann-Horn F, Schleip R (2012) The role of fibrosis in Duchenne muscular dystrophy. Acta Myologica 31(3):184 23620650 PMC3631802

[7] Aartsma-Rus A, Van Deutekom JC, Fokkema IF, Van Ommen GJB, Dunnen D, J.T (2006) Entries in the Leiden Duchenne muscular dystrophy mutation database: an overview of mutation types and paradoxical cases that confirm the reading‐frame rule. Muscle Nerve: Official J Am Association Electrodiagn Med 34(2):135–144 10.1002/mus.20586 16770791

[8] Tran VK, Nguyen NL, Tran LNT, Le PT, Tran AH, Pham TL, Lien NTK, Xuan NT, Thanh LT, Ta TV, Tran TH (2023) Merosin-deficient congenital muscular dystrophy type 1a: detection of LAMA2 variants in Vietnamese patients. Front Genet 14:p1183663 10.3389/fgene.2023.1183663 PMC10301838 37388928

[9] Zambon AA, Muntoni F (2021) Congenital muscular dystrophies: What is new? Neuromuscul Disord 31(10):931–942 34470717 10.1016/j.nmd.2021.07.009

[10] Jones KJ, Morgan G, Johnston H, Tobias V, Ouvrier RA, Wilkinson I, North KN (2001) The expanding phenotype of laminin α2 chain (merosin) abnormalities: case series and review. J Med Genet 38(10):649–657 11584042 10.1136/jmg.38.10.649 PMC1734735

[11] Gilhuis HJ, ten Donkelaar HJ, Tanke RB, Vingerhoets DM, Zwarts MJ, Verrips A, Gabreëls FJ (2002) Nonmuscular involvement in merosin-negative congenital muscular dystrophy. Pediatr Neurol 26(1):30–36 11814732 10.1016/s0887-8994(01)00352-6

[12] Durbeej M (2015) Laminin-α2 chain-deficient congenital muscular dystrophy: pathophysiology and development of treatment. Curr Top Membr 76:31–60 26610911 10.1016/bs.ctm.2015.05.002

[13] Philpot J, Bagnall A, King C, Dubowitz V, Muntoni F (1999) Feeding problems in merosin deficient congenital muscular dystrophy. Arch Dis Child 80(6):542–547 10332004 10.1136/adc.80.6.542 PMC1717951

[14] Geranmayeh F, Clement E, Feng LH, Sewry C, Pagan J, Mein R, Abbs S, Brueton L, Childs AM, Jungbluth H, De Goede CG (2010) Genotype–phenotype correlation in a large population of muscular dystrophy patients with LAMA2 mutations. Neuromuscul Disord 20(4):241–250 20207543 10.1016/j.nmd.2010.02.001

[15] Wallgren-Pettersson C, Bushby K, Mellies U, Simonds A (2004) 117th ENMC workshop: ventilatory support in congenital neuromuscular disorders—congenital myopathies, congenital muscular dystrophies, congenital myotonic dystrophy and SMA (II) 4–6 April 2003, Naarden, The Netherlands. Neuromuscul Disord 14(1):56–69 14659414 10.1016/j.nmd.2003.09.003

[16] Ahmad R, Naeem M (2025) A systematic review of hereditary neurological disorders diagnosed by whole exome sequencing in Pakistani population: updates from 2014 to November 2024. Neurogenetics 26(1):40 40178666 10.1007/s10048-025-00819-6

[17] Efthymiou S, Salpietro V, Malintan N, Poncelet M, Kriouile Y, Fortuna S, De Zorzi R, Payne K, Henderson LB, Cortese A, Maddirevula S (2019) Biallelic mutations in neurofascin cause neurodevelopmental impairment and peripheral demyelination. Brain 142(10):2948–2964 31501903 10.1093/brain/awz248 PMC6763744

[18] Ahmad R, Zamani M, Naeem M, Houlden H (2025) Rare autosomal recessive hereditary sensory and autonomic neuropathy type VI in a Pakistani family caused by a novel DST variant. Neurological Sciences, 1–8 10.1007/s10072-025-08424-z PMC12678596 40938507

[19] Ahmad R, Zamani M, Self E, Shah SUD, Naeem M, Houlden H (2025) Identification of a Novel GRM1 Frameshift Variant in Two Pakistani Families Broadens the Genetic Landscape of Ultra-Rare Spinocerebellar Ataxia Type 13. Cerebellum 24(5):145 40858856 10.1007/s12311-025-01897-w PMC12380963

[20] Quinodoz M, Peter VG, Bedoni N, Bertrand R, Cisarova B, Salmaninejad K, Sepahi A, Rodrigues N, Piran R, Mojarrad M, M. and, Pasdar A (2021) AutoMap is a high performance homozygosity mapping tool using next-generation sequencing data. Nat Commun 12(1):518 33483490 10.1038/s41467-020-20584-4 PMC7822856

[21] Hassan MJ, Mahmood S, Ali G, Bibi N, Waheed I, Rafiq MA, Ansar M, Ahmad W (2008) Intragenic deletions in the dystrophin gene in 211 Pakistani Duchenne muscular dystrophy patients. Pediatr Int 50(2):162–166 18353051 10.1111/j.1442-200X.2008.02538.x

[22] Ansar Z, Nasir A, Moatter T, Khan S, Kirmani S, Ibrahim S, Imam K, Ather A, Samreen A, Hasan Z (2019) MLPA analyses reveal a spectrum of dystrophin gene deletions/duplications in Pakistani patients suspected of having Duchenne/Becker muscular dystrophy: a retrospective study. Genetic Test Mol biomarkers 23(7):468–472 10.1089/gtmb.2018.0262 31157985

[23] Zehravi M, Wahid M, Ashraf J, Fatima T (2021) Whole-Exome Sequencing Identifies Small Mutations in Pakistani Muscular Dystrophy Patients. Genetic Test Mol Biomarkers 25(3):218–226 10.1089/gtmb.2020.0246 33734897

[24] Saredi S, Gibertini S, Matalonga L, Farina L, Ardissone A, Moroni I, Mora M (2019) Exome sequencing detects compound heterozygous nonsense LAMA2 mutations in two siblings with atypical phenotype and nearly normal brain MRI. Neuromuscul Disord 29(5):376–380 31040037 10.1016/j.nmd.2019.04.001

[25] Pagel KA, Pejaver V, Lin GN, Nam HJ, Mort M, Cooper DN, Sebat J, Iakoucheva LM, Mooney SD, Radivojac P (2017) When loss-of-function is loss of function: assessing mutational signatures and impact of loss-of-function genetic variants. Bioinformatics 33(14):i389–i398 28882004 10.1093/bioinformatics/btx272 PMC5870554

[26] Oliveira J, Santos R, Soares-Silva I, Jorge P, Vieira E, Oliveira ME, Moreira A, Coelho T, Ferreira JC, Fonseca MJ, Barbosa C (2008) LAMA2 gene analysis in a cohort of 26 congenital muscular dystrophy patients. Clin Genet 74(6):502–512 18700894 10.1111/j.1399-0004.2008.01068.x

[27] Muntoni F, Voit T (2004) The congenital muscular dystrophies in 2004: a century of exciting progress. Neuromuscul Disord 14(10):635–649 15351421 10.1016/j.nmd.2004.06.009

[28] Tubridy N, Fontaine B, Eymard B (2001) Congenital myopathies and congenital muscular dystrophies. Curr Opin Neurol 14(5):575–582 11562568 10.1097/00019052-200110000-00005

[29] Kirschner J, Bönnemann CG (2004) The congenital and limb-girdle muscular dystrophies: sharpening the focus, blurring the boundaries. Arch Neurol 61(2):189–199 14967765 10.1001/archneur.61.2.189

[30] de los Angeles Beytía M, Dekomien G, Hoffjan S, Haug V, Anastasopoulos C, Kirschner J (2014) High creatine kinase levels and white matter changes: clinical and genetic spectrum of congenital muscular dystrophies with laminin alpha-2 deficiency. Mol Cell Probes 28(4):118–122 24225367 10.1016/j.mcp.2013.11.002

[31] Dimova I, Kremensky I (2018) LAMA2 congenital muscle dystrophy: a novel pathogenic mutation in bulgarian patient. Case Rep Genet 2018(1):3028145 30147969 10.1155/2018/3028145 PMC6083551

[32] Oliveira J, Gruber A, Cardoso M, Taipa R, Fineza I, Gonçalves A, Laner A, Winder TL, Schroeder J, Rath J, Oliveira ME (2018) LAMA2 gene mutation update: Toward a more comprehensive picture of the laminin-α2 variome and its related phenotypes. Hum Mutat 39(10):1314–1337 30055037 10.1002/humu.23599

[33] Nguyen Q, Lim KRQ, Yokota T (2019) Current understanding and treatment of cardiac and skeletal muscle pathology in laminin-α2 chain-deficient congenital muscular dystrophy. The application of clinical genetics 113–130 10.2147/TACG.S187481 PMC6618038 31308722

[34] Yurchenco PD (2015) Integrating activities of laminins that drive basement membrane assembly and function. Curr Top Membr 76:1–30 26610910 10.1016/bs.ctm.2015.05.001

[35] Quijano-Roy S, Haberlova J, Castiglioni C, Vissing J, Munell F, Rivier F, Stojkovic T, Malfatti E, Gómez García de la Banda M, Tasca G, Costa Comellas L (2022) Diagnostic interest of whole-body MRI in early-and late-onset LAMA2 muscular dystrophies: a large international cohort. J Neurol 269(5):2414–2429 34559299 10.1007/s00415-021-10806-0

[36] Rini N, Lupica A, Alonge P, Crescimanno G, Pignolo A, Messina C, Paola S, Giuliano S, Borgione M, Lo E, Giudice M, Scuderi C (2025) Genetic and clinical spectrum of limb–girdle muscular dystrophies in Western Sicily. Genes 16(8):987 40870035 10.3390/genes16080987 PMC12386104

[37] Holmberg J, Durbeej M (2013) Laminin-211 in skeletal muscle function. Cell Adhes Migr 7(1):111–121 10.4161/cam.22618 PMC3544775 23154401

[38] Bushby K, Finkel R, Birnkrant DJ, Case LE, Clemens PR, Cripe L, Kaul A, Kinnett K, McDonald C, Pandya S, Poysky J (2010) Diagnosis and management of Duchenne muscular dystrophy, part 1: diagnosis, and pharmacological and psychosocial management. Lancet Neurol 9(1):77–93 19945913 10.1016/S1474-4422(09)70271-6

[39] Bitetti I, Mautone C, Bertella M, Manna MR, Varone A (2021) Early treatment with Ataluren of a 2-year-old boy with nonsense mutation Duchenne dystrophy. Acta Myologica 40(4):84 10.36185/2532-1900-062 PMC8744012 35047759

[40] Bladen CL, Salgado D, Monges S, Foncuberta ME, Kekou K, Kosma K, Dawkins H, Lamont L, Roy AJ, Chamova T, Guergueltcheva V (2015) The TREAT-NMD DMD Global Database: analysis of more than 7,000 Duchenne muscular dystrophy mutations. Hum Mutat 36(4):395–402 25604253 10.1002/humu.22758 PMC4405042

[41] Magri F, Govoni A, D’Angelo MG, Del Bo R, Ghezzi S, Sandra G, Turconi AC, Sciacco M, Ciscato P, Bordoni A, Tedeschi S (2011) Genotype and phenotype characterization in a large dystrophinopathic cohort with extended follow-up. J Neurol 258:1610–1623 21399986 10.1007/s00415-011-5979-z

[42] Garcia S, de Haro T, Zafra-Ceres M, Poyatos A, Gomez-Capilla JA, Gomez-Llorente C (2014) Identification of de novo mutations of Duchénnè/Becker muscular dystrophies in southern Spain. Int J Med Sci 11(10):988 25076844 10.7150/ijms.8391 PMC4115237

[43] Kesari A, Pirra LN, Bremadesam L, McIntyre O, Gordon E, Dubrovsky AL, Viswanathan V, Hoffman EP (2008) Integrated DNA, cDNA, and protein studies in Becker muscular dystrophy show high exception to the reading frame rule. Hum Mutat 29(5):728–737 18348289 10.1002/humu.20722

[44] Nakamura A, Shiba N, Miyazaki D, Nishizawa H, Inaba Y, Fueki N, Maruyama R, Echigoya Y, Yokota T (2017) Comparison of the phenotypes of patients harboring in-frame deletions starting at exon 45 in the Duchenne muscular dystrophy gene indicates potential for the development of exon skipping therapy. J Hum Genet 62(4):459–463 27974813 10.1038/jhg.2016.152

[45] Nakamura A, Fueki N, Shiba N, Motoki H, Miyazaki D, Nishizawa H, Echigoya Y, Yokota T, Aoki Y, Takeda SI (2016) Deletion of exons 3 – 9 encompassing a mutational hot spot in the DMD gene presents an asymptomatic phenotype, indicating a target region for multiexon skipping therapy. J Hum Genet 61(7):663–667 27009627 10.1038/jhg.2016.28

